# *STAT4* rs7574865 G/T and *PTPN22* rs2488457 G/C Polymorphisms Influence the Risk of Developing Juvenile Idiopathic Arthritis in Han Chinese Patients

**DOI:** 10.1371/journal.pone.0117389

**Published:** 2015-03-17

**Authors:** Zhi-Dan Fan, Fei-Fei Wang, Hui Huang, Na Huang, Hui-Hui Ma, Yi-Hong Guo, Ya-Yuan Zhang, Xiao-Qing Qian, Hai-Guo Yu

**Affiliations:** Department of Rheumatology and Immunology, Nanjing Children’s Hospital Affiliated to Nanjing Medical University, No. 72 Guangzhou Road, Nanjing, Jiangsu Province 210008, China; Sudbury Regional Hospital, CANADA

## Abstract

Juvenile idiopathic arthritis (JIA) is a common autoimmune disease characterized by environmental influences along with several predisposing genes in the pathogenesis. The protein tyrosine phosphatase nonreceptor 22 (*PTPN22*) and signal transducer and activator of transcription factor 4 (*STAT4*) have been recognized as susceptibility genes for numerous autoimmune diseases. Associations of *STAT4* rs7574865 G/T and *PTPN22* (rs2488457 G/C and rs2476601 C/T) polymorphisms with JIA have repeatedly been replicated in several Caucasian populations. The aim of this study was to investigate the influence of three polymorphisms mentioned above on the risk of developing JIA in Han Chinese patients. Genotyping was performed on a total of 137 Chinese patients with JIA (JIA group) and 150 sex and age frequency-matched healthy volunteers (Control group). The single-nucleotide polymorphisms (SNP) were determined by using direct sequencing of PCR-amplified products. There were significant differences of *PTPN22* rs2488457 G/C and *STAT4* rs7574865 G/T polymorphisms between both groups. However, no significant difference was observed in distribution frequencies of *PTPN22* rs2476601 polymorphism. The association with the *PTPN22* rs2488457 G/C polymorphism remained significant in the stratifications by age at onset, ANA status, splenomegaly, lymphadenectasis and involvement joints. As with the *STAT4* rs7574865 G/T polymorphisms, the enthesitis-related arthritis and presence of hepatomegaly had strong effect on the association. Our data strengthen *STAT4* rs7574865 G/T and *PTPN22* rs2488457 G/C polymorphisms as susceptibility factors for JIA.

## Introduction

Juvenile idiopathic arthritis (JIA) is one of the most common autoimmune diseases with genetic background and characterized by chronic inflammation of one or more joints in children [1–2]. It is of unknown etiology with onset before 16 years of age [3]. JIA encompasses seven subtypes of arthritis according to International League of Associations for Rheumatology (ILAR) classification system and causes functional disability and blinding eye disease [4]. Its prevalence varies worldwide among different ethnic and geographically distinct populations, underscoring the hereditary basis of the immunopathogenesis.

Protein tyrosine phosphatase nonreceptor 22 (*PTPN22*), a non-HLA gene located in chromosome 1p13.3-13.1, encodes lymphoid protein tyrosine phosphatase, which negatively regulates the T cells [5]. Previous studies found that *PTPN22* mutation may promote T cell activation and thus induce autoimmune diseases [6–7]. The presence of a C-T substitution (rs2476601) in the 14th exon of *PTPN22* gene, increases susceptibility to JIA in UK [8], Finnish [9] and Norwegian [10] populations, but presents no association in Greek [11] and Hungarian [2] patients. However, the rs2476601 SNP is almost absent in Asian populations [10, 12] and virtually non-polymorphic in Han Chinese according to HapMap data, suggesting that if *PTPN22* is associated with JIA in Chinese populations, then it is likely to be via another potentially functional SNP. In rheumatoid arthritis (RA), another SNP in *PTPN22* gene, a G-C substitution (rs2488457) in the promoter, had been addressed as a risk factor in several studies [10, 13]. As RA shares similar clinical presentation and pathological features with JIA [8, 14], so the rs2488457 SNP may confer susceptibility to the development of rheumatic diseases.

Signal transducer and activator of transcription factor 4 (*STAT4*) is another well-learned gene important for T cell differentiation [15]. The G-T substitution (rs7574865) in the intron 3 of *STAT4* has been identified as JIA susceptibility loci in UK [8] and non-Hispanic white [16] patients. A genome-wide association study (GWAS) conducted by Hinks et al [17] enhanced the role *STAT4* rs7574865 SNP in JIA susceptibility. Moreover, STAT4 is an important signaling molecule for IL-12, IL-23 and IFN-γ along with the IL-17 secretion [18–19]. So a functional SNP in *STAT4* may also possess potential for the development of JIA in Han Chinese patients.

We hypothesize that *STAT4* rs7574865 G/T and *PTPN22* rs2488457 G/C may also confer susceptibility to JIA in Han Chinese patients. Therefore, this study was performed to investigate the influence of three polymorphisms mentioned above on the risk of developing JIA in Han Chinese patients.

## Materials and Methods

### Study design

A case-control association study was performed to investigate whether the *STAT4* rs7574865 G/T and *PTPN22* (rs2488457 G/C and rs2476601 C/T) polymorphisms were associated with JIA. Stratification analysis was performed on the JIA cohort to investigate the effects of sex, age at onset (classified as age < 7 years or ≥ 7 years, based on previous study [20]), anti-nuclear antibody (ANA) status, hepatomegaly, splenomegaly, lymphadenectasis, wrist lesion, involvement joints (pauciarticular [≤4 joints] versus polyarticular [>4 joints]) and JIA subtype classified by ILAR criteria.

### Study subjects

One hundred and thirty seven JIA patients (78 males and 59 females) with a mean age of 8.41±3.17 years (range: 2–15 years) were consecutively recruited from the Affiliated Nanjing Children's Hospital of Nanjing Medical University between September 2010 and September 2012. A total of 150 sex and age frequency-matched healthy volunteers (86 males and 64 females) with a mean age of 8.10±2.81 years (range: 2–15 years) were included in the current study as a control group. The diagnosis and classification of JIA were made according to the ILAR criteria [4]. All patients and volunteers were Han Chinese. Patients were invited to participate without an attempt to select them by known or perceived risk factors. The study was approved by the Ethics Committee of Nanjing Medical University (Nanjing, China). Written informed consent on the use of clinical specimens for medical research was obtained before study entry from the next of kin, caretakers, or guardians on the behalf of the children participants involved in our study and adequate time to consider participation was given to the family. Each patient was interviewed by trained personnel using a pre-tested questionnaire to obtain information on demographic data and related risk factors for JIA, summarized in Table 1.

**Table 1:** Patient demographics and clinical data in JIA and Controls.

Variable	Controls	JIA
n	150	137
age, mean±SD years	8.10±2.81	8.41±3.17
Sex (male/female)	86/64	78/59
Age at onset, mean±SD years	-	7.74±2.90
JIA subtype, n(%)		
Systemic arthritis	-	43(31.39)
Oligoarthritis	-	48(35.04)
RF-negative polyarthritis	-	15(10.95)
RF-positive polyarthritis	-	11(8.03)
Enthesitis-related arthritis	-	12(8.76)
Undifferentiated arthritis	-	8(5.84)
Symptom		
ANA-positive, n(%)	-	24(17.52)
Hepatomegaly, n(%)	-	53(38.69)
Splenomegaly, n(%)	-	29(21.17)
Lymphadenectasis, n(%)	-	49(35.77)
Wrist lesion,n(%)	-	25(18.25)
Involvement joints(>4), n(%)	-	37(27.01)

### Genotyping

Blood samples were collected into Vacutainer Tubes containing ethylenediamine tetra-acetic acid (EDTA). Genomic DNA from whole blood samples was extracted with the QIAamp DNA Blood Mini Kit (Qiagen, Hilden, Germany) according to the “blood and body fluid protocol” as recommended by the manufacturer. The DNA extractions were stored at −20°C until analyzed. The primers to detect *STAT4* rs7574865 G/T and *PTPN22* (rs2488457 G/C and rs2476601 C/T) polymorphisms were described in Table 2, which were synthesized according to the previously published sequences by Genewiz Inc (Suzhou, China) [21–23]. The promoter region, exon 14 of *PTPN22* and intron 3 of *STAT4* were amplified by PCR using genomic DNA obtained from whole blood. Polymerase chain reaction was performed in a total volume of 20 μl reaction mixture containing 50 ng of genomic DNA, 0.2μl TransStartTaq polymerase (TransGen Biotech, Beijing, China), 0.5μl dNTP, 0.5μl each primer, 2μl 10×Buffer and 15.3μl ddH_2_O. The polymerase chain reaction profile consisted of an initial melting step at 95°C for 5 minutes followed by 40 cycles of 95°C for 45 seconds, 58°C for 45 seconds and 72°C for 30 seconds, and an additional extension 72°C for 5 minutes in a thermal cycler (Bio-Rad). Purified products were sequenced on an ABI Prism 3730xl sequencer (Applied Biosystems, Foster City, CA, USA) using BigDye Terminator Sequencing Standards. Gene-mapping software (GeneMapper, version 3.0) was employed for automated allele calling and manual verification. Ambiguous base calls were manually corrected by inspecting the sequence electropherograms. For quality control, repeated analyses were undertaken on 10% of randomly selected samples.

**Table 2:** Sequences of primers used for systematic search for SNPs in PTPN22 and STAT4.

SNP/rs number	primer	sequence
*PTPN22* rs2488457 G/C[22]	Forward	5’-AGAAAGCCTGAAGAACTG-3’
	Reverse	5’- ACCCATTGAGAGGTTATGCGAGCT-3’
*PTPN22* rs2476601 C/T[21]	Forward	5’-TCACCAGCTTCCTCAACCACA-3’
	Reverse	5’-GATAATGTTGCTTCAACGGAATTTA-3’
*STAT4* rs7574865 G/T[23]	Forward	5’-TTATGGAAAATTACATGAGTGTG-3’
	Reverse	5’-GCAAATCTTTGTAAAAAGTCAA-3’

### Statistical methods

Data analysis was done by SPSS statistical package version 16.0. Measurement data were expressed as the mean ± standard deviation (SD), and categorical data were expressed as frequency and percentage. The Student t test and Chi-square (χ2) were employed to compare the measurement data and categorical data, respectively. Odds ratio (OR) and 95% confidence interval (CI) were calculated for risk estimation. Chi-square (χ2) test was performed to assess deviation from Hardy-Weinberg equilibrium. A value of *P*<0.05 was considered statistically significant.

## Results

### Genotype and allele associations

The 287 DNA samples from 137 JIA patients and 150 controls were successfully genotyped and the concordance rates of repeated analyses were 100% for all SNPs in all subjects. Allele and genotype frequencies for the *PTPN22* SNPs and *STAT4* SNP were in Hardy-Weinberg equilibriumin in both the JIA patients and controls (*P*>0.05). The genotype distributions and allele frequencies of *STAT4* rs7574865 G/T and *PTPN22* (rs2488457 G/C and rs2476601 C/T) polymorphisms are illustrated in Table 3.

**Table 3:** Genotypic and allelic frequencies of three gene polymorphisms in JIA and Controls.

Region	SNP/rs number	JIA		Controls	
		Genotypes, n (%)	Allele, n (%)	Genotypes, n (%)	Allele, n (%)
Promoter	*PTPN22* rs2488457	GG	G	GG	G
		62(45.26)*	183(66.79) *	96(64.00)	239(79.67)
		GC	C	GC	C
		59(43.07) *	91(33.21) *	47(31.33)	61(20.33)
		CC		CC	
		16(11.68) *		7(4.67)	
Exon 14	*PTPN22* rs2476601	CC	C	CC	C
		137(100)	274(100)	150(100)	300(100)
		CT	T	CT	T
		0	0	0	0
		TT		TT	
		0		0	
Intron 3	*STAT4* rs7574865	GG	G	GG	G
		45(32.85) *	159(58.03) *	70(46.67)	203(67.67)
		TG	T	TG	T
		69(50.36)	115(41.97) *	63(42.00)	97(32.33)
		TT		TT	
		23(16.79)		17(11.33)	

*P<0.05 *vs*. healthy individuals Controls

The distribution frequencies of C allelotype (GC+CC genotype) and C allele of *PTPN22* rs2488457 in JIA patients were significantly higher than in Controls (*P*<0.05). No significant difference was observed in distribution frequencies of *PTPN22* rs2476601 polymorphism and allele between patients with JIA and healthy individuals. There were lower distribution frequencies of GG genotype and G allele of *STAT4* rs7574865 in JIA patients versus Controls (*P*<0.05).

### Associations between three gene polymorphisms and risk of JIA

The *PTPN22* rs2488457 C allele was associated with the risk of JIA in terms of the frequency of allele comparison (Table 4). When the *PTPN22* rs2488457 GG genotype was used as the reference, the GC or CC or GC/CC genotypes were all associated with the risk for JIA. In the recessive model, when the *PTPN22* rs2488457 GG/GC genotypes were used as the reference, the CC genotype was associated with susceptibility to JIA. However, no association of *PTPN22* rs2476601 C/T polymorphism with the risk for JIA was observed in this study.

**Table 4:** Associations between gene polymorphisms and risk of JIA.

SNP/rs number	Controls, n (%)	JIA, n (%)	OR(95%CI)	P values
*PTPN22* rs2488457				
GG	96(64.00)	62(45.26)	1.000	
GC	47(31.33)	59(43.07)	**1.944(1.180–3.201)**	**0.009**
CC	7(4.67)	16(11.68)	**3.539(1.377–9.905)**	**0.006**
GC+CC	54(36.00)	75(54.74)	**2.151(1.339–3.453)**	**0.001**
GG+GC	143(95.33)	121(88.32)	1.000	
CC	7(4.67)	16(11.68)	**2.701(1.076–6.782)**	**0.029**
G allele	239(79.67)	183(66.79)	1.000	
C allele	61(20.03)	91(33.21)	**1.948(1.336–2.841)**	**0.000**
*STAT4* rs7574865				
GG	70(46.67)	45(32.85)	1.000	
GT	63(42.00)	69(50.36)	**1.704(1.026–2.828)**	**0.039**
TT	17(11.33)	23(16.79)	**2.105(1.014–4.368)**	**0.044**
GT+TT	80(53.33)	92(67.15)	**1.789(1.107–2.890)**	**0.017**
GG+GT	133(88.67)	114(83.21)	1.000	
TT	17(11.33)	23(16.79)	1.578(0.804–3.100)	0.183
G allele	203(67.67)	159(58.03)	1.000	
T allele	97(32.33)	115(41.97)	**1.514(1.077–2.128)**	**0.017**

Bold values are statistically significant (*P*<0.05).

For *STAT4* rs7574865 polymorphism, when the GG genotype was used as the reference, the GT or TT or GT/TT genotypes were all associated with susceptibility to JIA. In the recessive model, when the GG/GT genotypes were used as the reference, the TT genotype was unassociated with susceptibility to JIA. The T allele was associated with the risk of JIA in terms of the frequency of allele comparison (Table 4). Multivariate logistic regression showed that *PTPN22* rs2488457 and *STAT4* rs7574865 were independent risk factors for JIA (Table 5).

**Table 5:** Analysis of independent risk factors for JIA by Multivariate logistic regression.

Variables	B	Std. Error	Wald	df	*P*	Exp(B)	95%CI for Exp(B)
Intercept	-0.534	0.203	6.881	1.000	0.009		
*PTPN22* rs2488457[GGvsGC/CC]	0.743	0.244	9.281	1.000	**0.002**	**2.102**	**1.303–3.389**
*STAT4* rs7574865[GGvsGT/TT]	0.550	0.249	4.879	1.000	**0.027**	**1.733**	**1.064–2.821**

Patient demographics and clinical data of JIA with normal and mutant gene

In terms of *PTPN22* rs2488457 SNP, there were more cases with ANA-positive status, splenomegaly, lymphadenectasis or involvement joints in GC/CC versus GG. Compared with GG genotype in *STAT4* rs7574865 SNP, the GT/TT patients presented more commonly with hepatomegaly (Table 6).

**Table 6:** Patient demographics and clinical data of JIA with normal and mutant gene.

Variable	*PTPN22* rs2488457			*STAT4* rs7574865		
	GG	GC+CC	P values	GG	GT+TT	P values
n	62	75		45	92	
age, mean±SD years	8.13±3.36	8.63±3.00	0.361	7.98±2.99	8.61±3.25	0.277
Sex (male/female)	36/26	42/33	0.808	21/24	57/35	0.090
Onset age, mean±SD years	7.43±2.99	7.98±2.82	0.267	7.22±2.65	7.99±2.99	0.149
JIA subtype, n(%)			0.552			0.235
Systemic arthritis	23(53.49)	20(46.51)		14(32.56)	29(67.44)	
Oligoarthritis	19(39.58)	29(60.42)		11(22.92)	37(77.08)	
RF-negative polyarthritis	8(53.33)	7(46.67)		5(33.33)	10(66.67)	
RF-positive polyarthritis	4(36.36)	7(63.64)		4(36.36)	7(63.64)	
Enthesitis-related arthritis	6(50.00)	6(50.00)		7(58.33)	5(41.67)	
Undifferentiated arthritis	2(25.00)	6(75.00)		4(50.00)	4(50.00)	
Symptom, n(%)						
ANA-positive	**3**(12.50)	**21**(87.50)	**0.000**	5(20.83)	19(79.17)	0.168
Hepatomegaly	19(35.85)	34(64.15)	0.079	**12**(22.64)	**41**(77.36)	**0.043**
Splenomegaly	**5**(17.24)	**24**(82.76)	**0.001**	7(24.14)	22(75.86)	0.261
Lymphadenectasis	**14**(28.57)	**35**(71.43)	**0.003**	14(28.57)	35(71.43)	0.427
Wrist lesion, n(%)	12(48.00)	13(52.00)	0.760	5(20.00)	20(80.00)	0.130
Involvement joints(>4), n(%)	**10**(27.03)	**27**(72.97)	**0.009**	10(27.03)	27(72.97)	0.378

Bold values are statistically significant (*P*<0.05).

Stratification analyses of *PTPN22* rs2488457 G/C polymorphism and risk for JIA

The association with the *PTPN22* rs2488457 G/C polymorphism was strong in JIA cases with an older age at onset and in those with ANA-positive status, splenomegaly, lymphadenectasis or more involvement joints (Table 7). Little difference in the risk was observed when cases were stratified by sex, hepatomegaly, wrist lesion or JIA subtype.

**Table 7:** Stratified analyses between *PTPN22* rs2488457 G/C polymorphism and the risk of JIA.

Variable	GG [*n*(%)]	GC+CC [*n*(%)]	OR(95%CI)	P values
Sex			1.088(0.551–2.147)	0.059
Male	36(46.2)	42(53.8)		
Female	26(44.1)	33(55.9)		
Age at onset (years)			**2.145(1.077–4.272)**	**0.029**
<7	33(55.9)	26(44.1)		
≥7	29(37.2)	49(62.8)		
ANA status			**7.648(2.159–27.092)**	**0.000**
Negative	59(52.2)	54(47.8)		
Positive	3(12.5)	21(87.5)		
Hepatomegaly			1.174(0.503–2.739)	0.711
Yes	16(55.2)	13(44.8)		
No	43(51.2)	41(48.8)		
Splenomegaly			**0.186(0.066–0.525)**	**0.001**
Yes	5(17.2)	24(82.8)		
No	57(52.8)	51(47.2)		
Lymphadenectasis			**0.333(0.158–0.705)**	**0.000**
Yes	14(28.6)	35(71.4)		
No	48(54.5)	40(45.5)		
Wrist lesion			1.145(0.480–2.728)	0.760
Yes	12(48.0)	13(52.0)		
No	50(44.6)	62(55.4)		
Involvement joints			**0.009(0.150–0.780)**	**0.009**
>4	10(27.0)	27(73.0)		
≤4	52(52.0)	48(48.0)		
JIA subtype				
Systemic arthritis	23(53.5)	20(46.5)	1.533(0.740–3.179)	0.249
Oligoarthritis	19(39.6)	29(60.4)	0.853(0.423–1.721)	0.657
RF-negative polyarthritis	8(53.3)	7(46.7)	1.418(0.484–4.158)	0.523
RF-positive polyarthritis	4(36.4)	7(63.6)	0.700(0.195–2.507)	0.582
Enthesitis-related arthritis	6(50.0)	6(50.0)	1.232(0.377–4.031)	0.730
Undifferentiated arthritis	2(25.0)	6(75.0)	0.406(0.079–2.083)	0.266

Bold values are statistically significant (*P*<0.05).

Stratification analyses of *STAT4* rs7574865 G/T polymorphism and risk for JIA

As with the *STAT4* rs7574865 G/T polymorphisms, presence of hepatomegaly and enthesitis-related arthritis had strong effect on the association (Table 8). Little difference in the risk was observed when cases were stratified by sex, age at onset, ANA status, splenomegaly, lymphadenectasis, wrist lesion, involvement joints or other JIA subtype.

**Table 8:** Stratified analyses between *STAT4* rs7574865 G/T polymorphisms and the risk of JIA.

Variable	GG[*n*(%)]	TG+TT[*n*(%)]	OR(95%CI)	P values
Sex			0.537(0.261–1.105)	0.090
Male	21(26.9)	57(73.1)		
Female	24(40.7)	35(59.3)		
Age at onset (years)			1.422(0.694–2.915)	0.336
<7	22(37.3)	37(62.7)		
≥7	23(29.5)	55(70.5)		
ANA status			2.030(0.704–5.584)	0.184
Negative	39(34.8)	73(65.2)		
Positive	5(20.8)	19(79.2)		
Hepatomegaly			**0.452(0.208–0.985)**	**0.043**
Yes	12(22.6)	41(77.6)		
No	33(39.3)	51(60.7)		
Splenomegaly			0.586(0.229–1.497)	0.261
Yes	7(24.1)	22(75.9)		
No	38(35.2)	70(64.8)		
Lymphadenectasis			0.735(0.344–1.570)	0.427
Yes	14(28.6)	35(71.4)		
No	31(35.2)	57(64.8)		
Wrist lesion			0.450(0.157–1.290	0.130
Yes	5(20.0)	20(80.0)		
No	40(35.7)	72(64.3)		
Involvement joints			0.688(0.299–1.583)	0.378
>4	10(27.0)	27(73.0)		
≤4	35(35.0)	65(65.0)		
JIA subtype				
Systemic arthritis	14(32.6)	29(67.4)	0.981(0.455–2.117)	0.961
Oligoarthritis	11(22.9)	37(77.1)	0.481(0.217–1.068)	0.069
RF-negative polyarthritis	5(33.3)	10(66.7)	1.025(0.328–3.199)	0.966
RF-positive polyarthritis	4(36.4)	7(63.6)	1.185(0.328–4.277)	0.796
Enthesitis-related arthritis	7(58.3)	5(41.7)	**3.205(0.957–10.741)**	**0.049**
Undifferentiated arthritis	4(50.0)	4(50.0)	2.146(0.511–9.010)	0.287

Bold values are statistically significant (*P*<0.05).

## Discussion

The primary novel findings in the present study are that the *PTPN22* rs2488457 G/C polymorphism and *STAT4* rs7574865 G/T polymorphism were associated with the risk of JIA in Han Chinese patients. We replicated the findings of a previous association of *PTPN22* rs2488457 C allele and *STAT4* rs7574865 T allele with JIA. Predictably, no association of *PTPN22* rs2476601 polymorphism was observed in Han Chinese population.

Systemic JIA is a heterogeneous form of arthritis in childhood and represents 10–20% of JIA in the Caucasian populations of Northern America and Europe [24]. However, two previous studies in China suggested a very high frequency of systemic JIA, accounting for 27.96% (26/93) and 47.03% (95/202), respectively [20, 25]. Similarly, another study reported 17 systemic JIA out of 33 patients with the frequency of 51.52% in the Japanese population [26]. In our study, systemic JIA was the second most common subtype (31.39% of cases), more than the reported frequency in Europe. The above evidences led us to hypothesis that the frequency of systemic JIA was higher in Asian versus Europe. The varied environment and genetic background may explain this inconformity. The male/female ratio in this study was 1.32, different from the female predominance observed in Europe. This is because of the number of systemic JIA cases, where the male/female ratio is around 1:1.

Several studies have investigated various degrees of potential association of *PTPN22* rs2476601 C/T polymorphisms with JIA in Caucasian populations, from minimal or no effect to strong effect [8–9, 11, 27–28]. There were no carriers of the T allele in either group of our study, and therefore it was not associated with JIA in this population. The combing evidence indicated this risk factor might be restricted to some ethnic groups under different environmental exposure, and the rs2476601 SNP is hardly polymorphic in East Asian populations, in accord with the previous reports on latent autoimmune diabetes in adult Chinese Hans [11] and autoimmune-disease in Japanese [22]. Although similar associations of *PTPN22* rs2488457 SNP have been found in many other autoimmune diseases [10, 13, 22], few studies are available regarding the role of rs2488457 SNP in the genetic susceptibility to JIA. We found that the rs2488457 SNP were associated with an increased susceptibility to JIA. To our knowledge, it is the first time to report its association with JIA in Han Chinese population. We find it intriguing that the rs2488457 SNP, in contrast to the rs2476601 SNP, is both polymorphic and possibly functional in Han Chinese JIA patients. The results supported our hypothesis that there were other potentially functional variants in *PTPN22* influencing the risk of JIA in Han Chinese patients.

Interesting, when stratifying the association with *PTPN22* SNP by the ILAR subgroups, different subtypes with positive effect have been declared in two previous studies. Hinks et al [8] published that the RF-negative polyarthritis subgroup of JIA showed strong association with the *PTPN22* SNP. However, no association with seven ILAR subgroups was observed in Hungarian JIA patients [2], consistent with our study. Regional variation in incidence and subtype ratio of JIA may account for the divergent results. It should be noted that we cannot exclude an association of this SNP with JIA subtype due to the relatively low sample size. The inconsistent results highlighted the importance of comparative studies in different ethnic populations. Hinks et al [8] found an association between the *PTPN22* SNP and the ANA status.

Similarly, we confirmed the strong association with the rs2488457 SNP in patients with ANA-positive status. It is unclear why the ANA status is associated with *PTPN22* SNP, but may be a mechanism of regulating the generation of disease-associated autoantibodies. One example was the discovery of *Ptpn8*, the mouse ortholog of the *PTPN22* gene, which may influence the generation of disease-associated autoantibodies [8]. Evidently, the serum levels of antibodies were elevated in *Ptpn8*-knockout mouse [29]. Thus, it has been speculated that *PTPN22* may be connected with the generation of disease-associated autoantibodies and may thereby contribute to development of JIA. Since older age at onset implies a longer environmental exposure, it may make sense that association with the rs2488457 G/C polymorphism was strong in JIA cases with an older age at onset. Based on strong associations with the rs2488457 SNP in patients with splenomegaly, lymphadenectasis or more involvement joints, we therefore confirmed the functional role of this polymorphism in the disease onset and progression of JIA in Han Chinese patients.

However, the role of *STAT4* SNP in the development of JIA is inconsistent, due to the remarkably varied risk reported previously in different ethnic populations [11, 14, 29]. A genome-wide association study (GWAS) with largest collection of JIA cases confirmed a role of *STAT4* rs7574865 SNP in JIA susceptibility [17]. We replicated the association of rs7574865 SNP with JIA, in accordance to the recent data [29], suggesting a possible functional role in JIA. Unlike the rs2488457 SNP, we found the presence of hepatomegaly and enthesitis-related arthritis had strong effect on the association. The difference just reached statistical significance due to a small sample size of enthesitis-related arthritis, so it still needed the support from large-size cohorts. Interesting, we failed to found the rs7574865 SNP, in contrast to the rs2488457 SNP, was strongly associated with the age at onset in Han Chinese JIA patients. Different clinical feathers are associated with the SNP within different genes, reminding us that *PTPN22* rs2488457 G/C and *STAT4* rs7574865 G/T polymorphisms may contribute differently to the etiology of JIA.

Few international cohorts had made the stratified analyses between identified genetic loci and the risk of JIA, so limited information can be instructive for clinical investigation. A definite advantage of our study was the attention paid on the stratification analysis to investigate the SNP association in different subgroups. The genotypes of examined polymorphisms satisfied Hardy-Weinberg equilibrium in both patient’s cohort and control group, suggesting the results are unlikely to be biased by sampling. However, several limitations of the present study need to be addressed. Firstly, this study covered a relatively small sample size, and our findings need confirmation in larger patient cohorts. Secondly, a single-center study is not sufficient to fully interpret the association between polymorphisms and susceptibility to JIA. Inspiringly, the recent JIA Immunochip study completed the largest and most comprehensive genetic analysis of JIA in Europeans to date, which showed association of both *PTPN22* and *STAT4* with JIA [17]. Since the pathogenesis of JIA is highly complex, involving genetic background and environmental conditions, further studies with larger sample sizes on a multi-center level are recommended.

Taken together, our data strengthen *STAT4* rs7574865 G/T and *PTPN22* rs2488457 G/C polymorphisms as susceptibility factors for JIA and provide further evidence for a common origin of autoimmune diseases. In consideration of relatively small number of patients evaluated and gene-environment interactions, other candidate SNPs in the *PTPN22* and *STAT4* genes will still need to be evaluated to conclusively elucidate the directly involved genetic polymorphism.
